# INTERPRETING PERSON-CENTEREDNESS IN RELATION TO CARE AND THE WORKFORCE FOR OLDER ADULTS

**DOI:** 10.1093/geroni/igad104.3438

**Published:** 2023-12-21

**Authors:** Antonio Saavedra, Philip Sloane, Sheryl Zimmerman, Sam Fazio

**Affiliations:** University of North Texas Health Science Center, Fort Worth, Texas, United States; University of North Carolina, Chapel Hill, North Carolina, United States; University of North Carolina, Chapel Hill, North Carolina, United States; Alzheimer’s Association, Chicago, Illinois, United States

## Abstract

The concept of person-centeredness has been widely referenced and seemingly adopted as a gold standard practice, especially in relation to care for older persons. However, the concept of person-centeredness has evolved over time, resulting in a lack of definitional clarity. As part of a series of studies exploring person-centeredness, we investigated the meaning of person-centeredness as it relates to care and the workforce for older adults. Six think-tank meetings (two in-person; four virtual) were convened, including a national purposive sample of 33 participants selected for diversity and expertise, and including providers, consumers, policy makers, and researchers. Qualitative methods were used to analyze notes and transcripts, resulting in four overarching themes: (1) Different care settings (e.g., hospice, nursing home) tend to vary in the extent and manner of person-centeredness due to their inherent characteristics and goals of care. (2) Leadership tends to assign priority to quality citations and safety protocols, which creates dissonance between the desire to be more person-centered and a demanding organizational culture. (3) Person-centeredness can be conveyed through concrete practices (e.g., care for activities of daily living) or general principles; the former may be easier to implement. (4) The lack of an operationalized person-centeredness definition has created a gap in how to teach it. This presentation will tie together the four themes and address attitudes and approaches within organizations and toward care providers.

